# A comparative analysis of clinical outcomes in hematological patients afflicted with bacteremia attributable to carbapenem-resistant *Klebsiella pneumoniae* versus *Escherichia coli*

**DOI:** 10.3389/fcimb.2025.1600746

**Published:** 2025-06-17

**Authors:** Jiali Wang, Jia Liu, Qingsong Lin, Fengkui Zhang, Yizhou Zheng, Zhijian Xiao, Jianxiang Wang, Aiming Pang, Yi He, Erlie Jiang, Sizhou Feng, Mingzhe Han, Weihua Zhai

**Affiliations:** ^1^ State Key Laboratory of Experimental Hematology, National Clinical Research Center for Blood Diseases, Haihe Laboratory of Cell Ecosystem, Institute of Hematology & Blood Diseases Hospital, Chinese Academy of Medical Sciences & Peking Union Medical College, Tianjin, China; ^2^ Tianjin Institutes of Health Science, Tianjin, China; ^3^ State Key Laboratory of Trauma, Burns and Combined Injury, Medical Center of Hematology, The Second Affiliated Hospital of Army Medical University, Key Subject of Chongqing, Chongqing, China

**Keywords:** clinical outcomes, carbapenem-resistant *Klebsiella pneumoniae*, carbapenem resistant *Escherichia coli*, bacteremia, hematological patients

## Abstract

**Introduction:**

Carbapenem-resistant Enterobacterales (CRE) bloodstream infections (BSI) represent a frequent and grave complication among hematological patients, whose prevailing culprits are Carbapenem-Resistant Klebsiella pneumoniae (CRKP) and Escherichia coli bacteremia (EC). Nevertheless, there is a paucity of studies that have undertaken a comparative analysis of clinical outcomes in patients afflicted with CRKP and EC.

**Methods:**

This study was conducted with the aim of identifying the microbiological and clinical characteristics of hematological patients suffering from bacteremia caused by CRKP and CREC.

**Results:**

The cohort included 90 patients with equal proportions of CRKP BSI and CREC BSI from 2017 to 2022. Among the tested CRE strains (n = 45) for carbapenemase (CP) genes, the KPC gene was most commonly found in CP-CRKP isolates (12/21), while the NDM gene predominated among CP-CREC strains (18/24). A comparison of drug susceptibility showed that CREC was significantly more susceptible to tigecycline than CRKP (97.73% vs. 64.86%, P = 0.018). Patients treated with tigecycline-based therapy had a higher survival rate in the CREC group (18/24,75%) compared to the CRKP group (8/14,57.1%). The CRKP group had a significantly lower rate of prior cephalosporin use within 30 days compared to the CREC group (27% vs. 49%, P = 0.03) and a higher incidence of multi-site infections before BSI (44% vs. 8.9%, P<0.001). Multivariate analysis showed that BSI caused by CRKP was an independent risk factor for survival (P = 0.029), while CAZ-AVI-based therapy emerged as an independent factor improving patient prognosis (P =0.013).

**Conclusions:**

Our results found that bacteremia instigated by CRKP was associated with a less favorable prognosis when compared to cases induced by CREC. Moreover, treatment regimens incorporating CAZ-AVI have the potential to enhance the prognosis of patients grappling with CRE BSI.

## 1 Introduction

The widespread usage of broad-spectrum antibiotics has led to a surge in the incidence of bloodstream infections (BSI) caused by multidrug-resistant Enterobacterales, notably carbapenem-resistant Enterobacterales (CRE), presenting a formidable global public health challenge (Logan and Weinstein, 2017; Lutgring, 2019). Patients with hematological malignancies who receive chemotherapy or immunosuppressive treatment, experience neutropenia, undergo invasive procedures, and receive carbapenem antibiotics are more susceptible to CRE BSI, with an incidence ranging from 16% to 24% and a crude mortality rate of approximately 60% (Satlin et al., 2013; Satlin et al., 2016). Differing from the patterns observed in Europe and the United States, Klebsiella pneumoniae (KP) and Escherichia coli (EC) approximately share an equal burden in China and are the predominant members of the Enterobacterales family, as highlighted by the CHINET surveillance (Chen et al., 2022). This regional distinction underscores the need to delve into the dissimilarities in virulence and enzyme production between CRKP and CREC, as these disparities can potentially exert a profound influence on clinical outcomes and guide antibiotic treatment decisions. Nevertheless, the current body of literature is conspicuously sparse in terms of information regarding the prognostic disparities between CRKP and CREC-induced bacteremia, particularly among hematological patients.

Thus, we undertook this study to delve into the epidemiology, clinical features, and outcomes of CRKP and CREC BSI in patients grappling with hematological malignancies. Our objective is to furnish essential data that can serve as the bedrock for advancing the management and enhancing the judicious utilization of antibiotics in the hematological patient population afflicted by CRE infections.

## 2 Materials and methods

### 2.1 Study design and patients

This retrospective cohort study was conducted at a tertiary hospital specialized in blood diseases, situated in Tianjin, China, boasting a bed capacity of 769. The hematology ward of this hospital annually admits nearly 30,000 patients. We gathered data from hematological patients who were hospitalized during the period spanning from February 1, 2017, to January 31, 2022. Inclusion criteria encompassed patients diagnosed with CRE BSI, with the exclusion of individuals under the age of 14, those concurrently infected with both CREC and CRKP, and those whose CRE BSI originated from strains other than CREC and CRKP. Patient follow-up extended for a duration of 30 days from the initial isolation of CRE from their blood cultures. It’s noteworthy that each case was counted only once, even in scenarios where multiple instances of CRE BSI were documented. The commencement date of BSI was precisely defined as the date of specimen collection when the initial positive blood culture was obtained. Clinical outcomes were evaluated independently by investigators who remained blinded to the variables under investigation.

This study received approval from the ethical committee of the Institute of Hematology and Blood Diseases Hospital, Chinese Academy of Medical Sciences. Given the retrospective and anonymized nature of the analysis, the requirement for written informed consent was waived by the ethics committee for health research. Furthermore, this study was conducted in strict adherence to the principles set forth in the Declaration of Helsinki.

### 2.2 Microbiology

Bacterial culture, identification, and drug sensitivity assessments were conducted within the microbiology laboratory, with a focus on the targeted bacteria, namely CREC and CRKP. All CRE strains were isolated exclusively from blood samples. Blood culture procedures were performed employing an automatic blood culture system (BD, USA). The isolation and identification of bacteria adhered strictly to the guidelines outlined in the National Clinical Laboratory Procedures. For the identification of isolates, the VITEK 2 Compact system (bioMérieux, France) was utilized, with further confirmation carried out through Matrix-Assisted Laser Desorption/Ionization Time-of-Flight Mass Spectrometry (MALDI-TOF MS, bioMérieux, France). Antibiotic susceptibility testing was executed within the hospital’s microbiology laboratory, employing an automated system - VITEK 2 Compact. A comprehensive panel of antibiotics was subjected to testing, encompassing penicillins (ticarcillin, piperacillin), β-lactamase inhibitor combinations (amoxicillin/clavulanic acid, piperacillin/tazobactam, cefoperazone/sulbactam), cephalosporins (cefazolin, cefuroxime, ceftazidime, cefepime, cefotaxime, cefotetan, cefpodoxime, ceftizoxime), quinolones (levofloxacin, moxifloxacin, ciprofloxacin, norfloxacin), carbapenems (imipenem, meropenem, doripenem, ertapenem), aminoglycosides (amikacin, tobramycin), tetracyclines (tigecycline, minocycline), aztreonam, trimethoprim/sulfamethoxazole, and. The determination of the minimum inhibitory concentration (MIC) was conducted in accordance with the guidelines provided in the 31st Edition of the Clinical and Laboratory Standards Institute (CLSI) M100-Performance Standards for Antimicrobial Susceptibility Testing (Clinical and Laboratory Standards Institute, USA. Performance standards for antimicrobial susceptibility testing, M100 30th edition;2020. Available from: https://clsi.org/standards/products/microbiology/documents/m100/. Accessed January 29, 2021). For the detection of carbapenemases within CRE, we employed the modified carbapenem inactivation methods (mCIM and eCIM) as specified in the CLSI 31st Edition. Genomic DNA was extracted from bacterial colonies by suspending them in phosphate-buffered saline (PBS) and heating at 100°C for 5 minutes. The suspensions were then centrifuged at 15,000 rpm for 1 minute, and the resulting supernatant was used as the template for PCR amplification. The presence of carbapenemase genes (blaKPC, blaNDM), were assessed using primer sets and PCR conditions described in prior studies (**Supplementary Table S2**) (Poirel et al., 2011). Furthermore, the rapid identification of carbapenemase was facilitated using colloidal gold immunochromatography by Carba-5 (Bodendoerfer et al., 2019).

### 2.3 Data Collection and definition

Comprehensive baseline characteristics were collected, encompassing demographic information such as age and gender, hematologic disease diagnoses, Charlson Comorbidity Index scores, antibiotic usage in the 30 days preceding CRE BSI, prior CRE colonization status, microbiological attributes including carbapenemase production and drug sensitivity results of isolates, treatment history encompassing chemotherapy, immunosuppressive therapy, and hematopoietic stem cell transplantation, complications such as mucositis, diarrhea, fungal infections, perianal infections, and bacteremia caused by other bacteria, as well as comorbidities other than infections. Additionally, data concerning the timing of agranulocytosis, the duration of hospitalization prior to the onset of BSI, antibiotic treatments, and survival for CRE infections were meticulously gathered.

CRE was defined as Enterobacterales bacteria that met any of the following criteria: 1) Displayed resistance to any carbapenems (with a minimum inhibitory concentration [MIC] of imipenem, meropenem, and doripenem ≥ 4 mg/L, or a MIC of ertapenem ≥ 2 mg/L). 2) Produced carbapenemase enzymes. 3) If naturally resistant to imipenem, as in the case of Morganella morganii, Proteus genus, or Providencia genus, it must also exhibit resistance to other carbapenems such as meropenem, ertapenem, and doripenem (Adeolu et al., 2016). The initiation of BSI was precisely defined as the date when the positive blood culture sample was collected. Prior 90-day CRE colonization refers to the detection of CRE strains in routine perianal and pharyngeal swab screenings prior to the occurrence of bloodstream infection, without any clinical signs of infection. Multi-site colonization refers to the detection of CRE strains in multiple open sites, such as perianal, pharyngeal, etc., without any clinical evidence of infection. Ceftazidime/Avibactam-based therapy was characterized as either Ceftazidime/Avibactam (CAZ-AVI) monotherapy or combination therapy involving CAZ-AVI (Zhang et al., 2023). Appropriate empirical therapy referred to the administration of *in vitro* active antimicrobials against the isolates before receiving a susceptibility report (Seo et al., 2020). Monotherapy signified a treatment regimen involving a single agent with *in vitro* activity, while combination therapy entailed the use of two or more agents with *in vitro* activity (Zhao et al., 2020). It’s important to note that all antimicrobial agents included in the analysis were administered for a minimum duration of 48 hours.

### 2.4 Statistical analysis

The statistical analysis was performed using R (version 4.0.4). The normal distribution of continuous data was assessed using the Shapiro-Wilk test. Continuous variables that followed a normal distribution were presented as means ± standard deviation (SD) and analyzed using the Student’s t-test. Non-normally distributed continuous variables were presented as medians (minimum, maximum) and analyzed using the Mann-Whitney U test. Categorical data were reported as numbers (n) and percentages (%) and analyzed using the Chi-square test or Fisher’s exact test. Variables with a *P*-value ≤0.05 in univariate analysis were included in multivariate logistic regression analysis. Missing data for the variable “prior 90-day CRE colonization” in multivariate analysis were handled through multiple imputations (100 cycles), and final results were obtained using the mice package. All statistical analyses employed two-tailed tests, with *P*-values less than 0.05 considered statistically significant. Survival curves were generated utilizing the Kaplan-Meier method.

## 3 Results

### 3.1 Patient demographics and epidemiological trends

**Figure 1:**
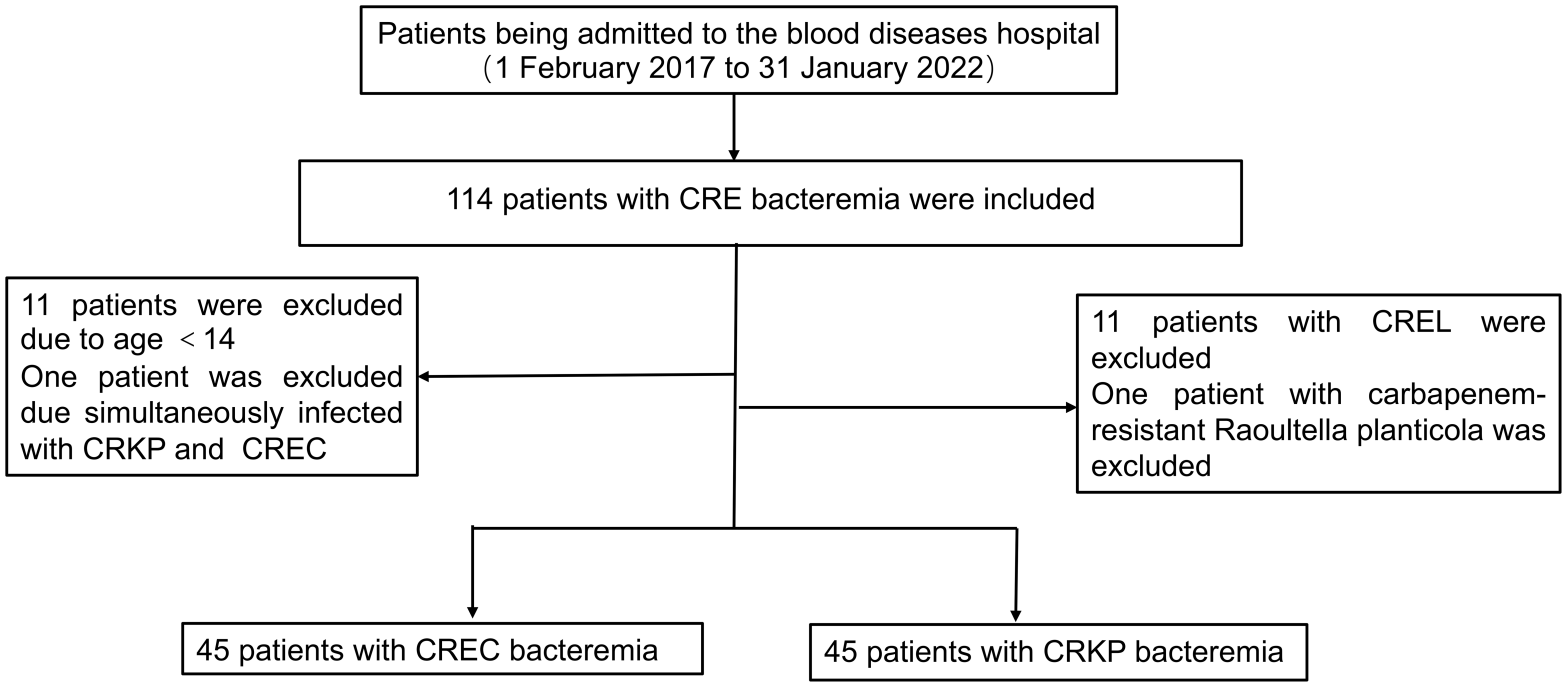
Participant flow diagram.

Over a span of five years, commencing on February 1, 2017, and concluding on January 31, 2022, our institution identified 114 individuals afflicted with hematologic disorders who developed CRE BSI. Adhering rigorously to predefined inclusion and exclusion criteria, our analysis encompassed a cohort of 90 patients comprising equal proportions of CRKP BSI and CREC BSI cases (**Figure 1**). From 2017 to 2021, the overall trend of CRE BSI cases exhibited an upward trajectory, with a subsequent plateau observed from 2019 to 2021. A notable observation in 2018 highlighted that the frequency of CRKP infections was double (12 vs 6) that of CREC infections, albeit the overall numbers of infections caused by these two pathogens were closely matched. CRKP and CREC emerged as the predominant pathogens in our facility, responsible for CRE BSIs, with their incidences showing negligible differences (**Supplementary Figure S1**). In 2019, CAZ-AVI was used to treat CRE BSI, resulting in a significant decrease in the annual 30-day mortality rate of CRE BSI thereafter (**Supplementary Figure S1**).

### 3.2 Microbiological profiles

Before October 2017, our hospital was unable to determine whether the CRE isolates produced carbapenemase. Prior to November 2019, our hospital laboratory could only identify if the CRE isolates were carbapenemase-producing but could not differentiate between serine-carbapenemases and metallo-carbapenemases. Consequently, out of the 90 patients included in this study, it was uncertain whether CRE isolates produced carbapenemases among 7 patients. Moreover, 66 (79.5%) were identified as carbapenemase-producing CRE (CP-CRE) isolates, and 17 (20.5%) were non-CP-CRE cases among the remaining 83 patients. Further detailed analysis was feasible for 45 of the 66 CP-CRE cases, employing PCR and colloidal gold immunochromatography tests to pinpoint genes responsible for carbapenemase production. Our findings reveal a predominance of metallo-carbapenemases among the CP-CRE isolates derived from our hospital’s patients. Specifically, the CP-CRE isolates from the CREC group almost universally produced metallo-carbapenemases. In contrast, approximately two-thirds of the CRKP isolates were identified as metallo-carbapenemase producers. Within the CP-CRKP contingent, the Klebsiella pneumoniae carbapenemase (KPC) gene was the most frequently identified (in 12 out of 21 cases), whereas the New Delhi metallo-β-lactamase (NDM) gene was predominantly observed in CP-CREC strains (18 out of 24 cases), as detailed in **Supplementary Table S1**.

A further comparison of the drug susceptibility of the two strains to common clinical antibiotics is shown in **Supplementary Figure S2**. Notably, CREC exhibited a markedly higher susceptibility to tigecycline compared to CRKP, with susceptibility rates of 97.73% versus 64.86%, respectively (P = 0.018). Despite the generally low susceptibility of both strains to moxifloxacin, CRKP demonstrated a relatively greater susceptibility than CREC, with rates of 22.33% versus 8.89%, respectively (P = 0.048). Contrasts in resistance patterns to meropenem and imipenem between the two bacterial species did not achieve statistical significance, as depicted in **Supplementary Figure S2**. Additionally, the degree of drug resistance, quantified by the MIC for meropenem or imipenem, was similarly comparable between the strains, as outlined in **Table 1**.

**Table 1:** Baseline characteristics and outcomes of patients and clinical isolates infected with carbapenem-resistant Klebsiella pneumoniae (CRKP) and carbapenem-resistant Escherichia coli (CREC) bacteria.

Characteristic	CREC(n=45)	CRKP(n=45)	P-value^1^
Sex, n (%)			0.389
Male	29 (64%)	25 (56%)	
Female	16 (36%)	20 (44%)	
Age, mean (min, max)	41 (17,64)	40 (16,71)	0.981
Disease			0.502
Aplastic anemia	4 (8.9%)	6 (13%)	
Hematological malignances	41 (91%)	39 (87%)	
CCI score, n (%)			0.502
0~2	41 (91%)	39 (87%)	
>2	4 (8.9%)	6 (13%)	
Prior 90-day CRE colonization, n (%)			0.915
No	18 (50%)	21 (51%)	
Yes	18 (50%)	20 (49%)	
Missing data	9	4	
Multi-site colonization, n (%)			>0.999
No	58 (92%)	26 (100%)	
Yes	5 (7.9%)	0 (0%)	
Missing data	8	5	
Carbapenemases production, n (%)			0.127
No	6 (14%)	11 (28%)	
Yes	37 (86%)	29 (72%)	
Missing data	2	5	
Peak procalcitonin (PCT), pg/mL, n (%)			0.529
0≤PCT<0.5	23 (61%)	19 (51%)	
0.5≤PCT<2	8 (21%)	10 (27%)	
2≤PCT<10	3 (7.9%)	6 (16%)	
PCT≥10	4 (11%)	2 (5.4%)	
Missing data	7	8	
Hematopoietic Stem Cell Transplantation, n (%)	12 (27%)	18 (40%)	0.180
Immunosuppressive therapy, n (%)	15 (33%)	18 (40%)	0.512
Chemotherapy, n (%)	40 (89%)	40 (89%)	>0.999
Comorbidity besides infection, n (%)	11 (24%)	13 (29%)	0.634
Multi-site infection before CRE BSI, n (%)	4 (8.9%)	20 (44%)	<0.001
Agranulocytosis time before BSI, mean (min, max)	15 (0,118)	37 (0,425)	0.357
Hospitalization time before BSI, mean (min, max)	32 (0,157)	41 (5,163)	0.057
Meropenem MIC of isolate>8ug/ml, n (%)	35 (78%)	37 (82%)	0.598
Imipenem MIC of isolate>8ug/ml, n (%)	34 (76%)	34 (76%)	>0.999
Prior 30-day glucocorticoids using, n (%)	25 (56%)	23 (51%)	0.673
Prior 30-day antibiotic using, n (%)			
BLBLIs	18 (40%)	20 (44%)	0.670
Cephalosporins	22 (49%)	12 (27%)	0.030
Linezolid	6 (13%)	13 (29%)	0.071
Peptide antibiotics	4 (8.9%)	10 (22%)	0.081
Aminoglycosides	3 (6.7%)	3 (6.7%)	>0.999
Tigecycline	7 (16%)	11 (24%)	0.292
Fluoroquinolones	1 (2.2%)	4 (8.9%)	0.361
Carbapenems	31 (69%)	31 (69%)	>0.999
Appropriate empirical therapy with 48h, n (%)	34 (76%)	32 (71%)	0.634
Ceftazidime/Avibactam based therapy ^2^, n (%)	16 (36%)	12 (27%)	0.362
Tigecycline-based therapy ^3^, n (%)	22 (49%)	20 (44%)	0.673
Colistin-based therapy ^4^, n (%)	1 (2.2%)	3 (6.7%)	0.616
Combined therapy, n (%)	11 (24%)	13 (29%)	0.634
Thirty-day mortality	8 (18%)	20 (44%)	0.006

^1^Pearson’s Chi-squared test; Wilcoxon rank sum test; Fisher’s exact test.

^2^Ceftazidime/Avibactam-based therapy was defined as Ceftazidime/Avibactam monotherapy and Ceftazidime/Avibactam combination therapy.

^3^Tigecycline-based therapy was defined as tigecycline monotherapy and tigecycline combination therapy without Ceftazidime/Avibactam.

^4^Colistin-based therapy was defined as colistin monotherapy and colistin combination therapy without Ceftazidime/Avibactam and Tigecycline.

### 3.3 Clinical and treatment characteristics

**Figure 2:**
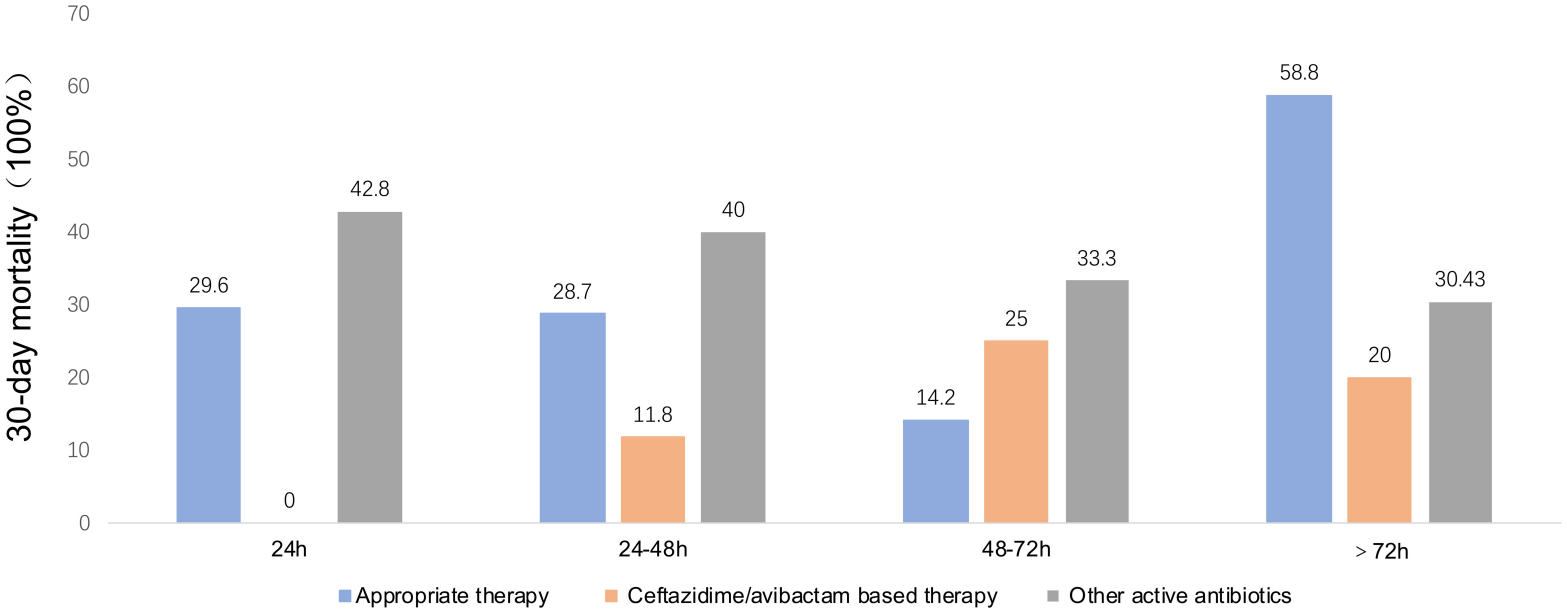
Thirty mortality rates by time from blood collection to appropriate therapy, ceftazidime/Avibactam-based therapy, and other active antibiotics. Other active antibiotics refer to antimicrobial agents that were active *in vitro* against the isolates, except for ceftazidime-avibactam.

The comparative analysis between patients infected by CRKP and those by CREC revealed no significant disparities in several clinical parameters including age, sex distribution, disease type, Charlson Comorbidity Index (CCI) scores, history of prior colonization, extensive antibiotic use within the preceding 30 days, prior therapeutic interventions, complications, concurrent infections, duration of agranulocytosis, peak procalcitonin (PCT) levels, and overall hospital stay. Noteworthy, the CRKP cohort displayed a considerably lower incidence of cephalosporin usage within the 30 days preceding infection diagnosis (27% vs. 49%, P = 0.03) and a higher prevalence of multi-site infections prior to BSI onset (44% vs. 8.9%, P <0.001). However, there were no differences between the two groups regarding the initial time to appropriate empirical therapy or drug combination for treatment based on CAZ-AVI and tigecycline regimens. Univariate analysis underscored a significantly elevated 30-day mortality rate among patients with CRKP infections compared to those with CREC infections (44% vs. 18%, P = 0.006) (**Table 1**). Out of the cohort, 80 patients were administered antibiotics deemed appropriate for their condition (**Table 2**). The correlation between therapeutic choices and patient outcomes was particularly pronounced in the context of drug susceptibility profiles. Specifically, the CREC group benefited more from tigecycline-based treatments, with a survival rate of 75% (18 out of 24 patients) compared to 57.1% in the CRKP group (8 out of 14 patients) (P>0.05). The analysis further revealed superior overall survival rates among patients receiving CAZ-AVI-based therapies over those treated with either tigecycline or polymyxin-based strategies. Furthermore, among patients who received CAZ-AVI-based therapy within 72 hours, i.e., at an earlier stage, 30-day mortality rates tended to be lower, although this difference did not reach statistical significance (**Figure 2**).

**Table 2:** Appropriate antibiotic treatments in patients with carbapenem-resistant Klebsiella pneumoniae (CRKP) and carbapenem-resistant Escherichia coli (CREC) bacteria.

Antimicrobial therapy	Total (30-day mortality)	CRKP (30-day mortality), N=38	CREC (30-day mortality), N=42
CAZ-AVI-based therapy	35 (4)	19 (4)	16 (0)
CAZ-AVI	1	1	
CAZ-AVI+ tigecycline	3 (1)	3 (1)	
CAZ-AVI+ amikacin	1	1	
CAZ-AVI+ cefoperazone-sulbactam	1 (1)	1 (1)	
CAZ-AVI+ATM	7	3	4
CAZ-AVI+ATM+ tigecycline	16 (1)	7 (1)	9
CAZ-AVI+ATM+ polymyxin	3 (1)	2 (1)	1
CAZ-AVI+ATM+ tigecycline+ polymyxin	2	1	1
CAZ-AVI+ATM+ etimicin	1		1
Tigecycline-based therapy	38 (12)	14 (6)	24 (6)
Tigecycline	11 (5)	5 (4)	6 (1)
Tigecycline+ polymyxin	3	1	2
Tigecycline+ ATM	2		2
Tigecycline+ etimicin	10 (3)	5 (1)	5 (2)
Tigecycline+ amikacin	1		1
Tigecycline+ carbapenems	1		1
Tigecycline+ cephalosporins	3 (3)	1 (1)	2 (2)
Tigecycline+ etimicin+ carbapenems	2	2	
Tigecycline+ etimicin+ polymyxin	2		2
Tigecycline+ etimicin+ cefoperazone-sulbactam	2		2
Tigecycline+ etimicin+ Moxifloxacin	1 (1)		1 (1)
Polymyxin -based therapy	4 (3)	3 (2)	1 (1)
Polymyxin+ etimicin	3 (3)	2 (2)	1 (1)
Polymyxin+ etimicin+ ATM	1	1	
Other active antibiotics	4 (2)	3 (2)	1 (0)
Etimicin+ carbapenems	2 (2)	2 (2)	
Etimicin+ ciprofloxacin + cefoperazone-sulbactam	1	1	
Carbapenems	1		1

CAZ-AVI, Ceftazidime/Avibactam; ATM, aztreonam.

Other active antibiotics refer to antimicrobial agents that were active *in vitro* against the isolates, except for ceftazidime-avibactam.

### 3.4 Risk factors for mortality

**Figure 3:**
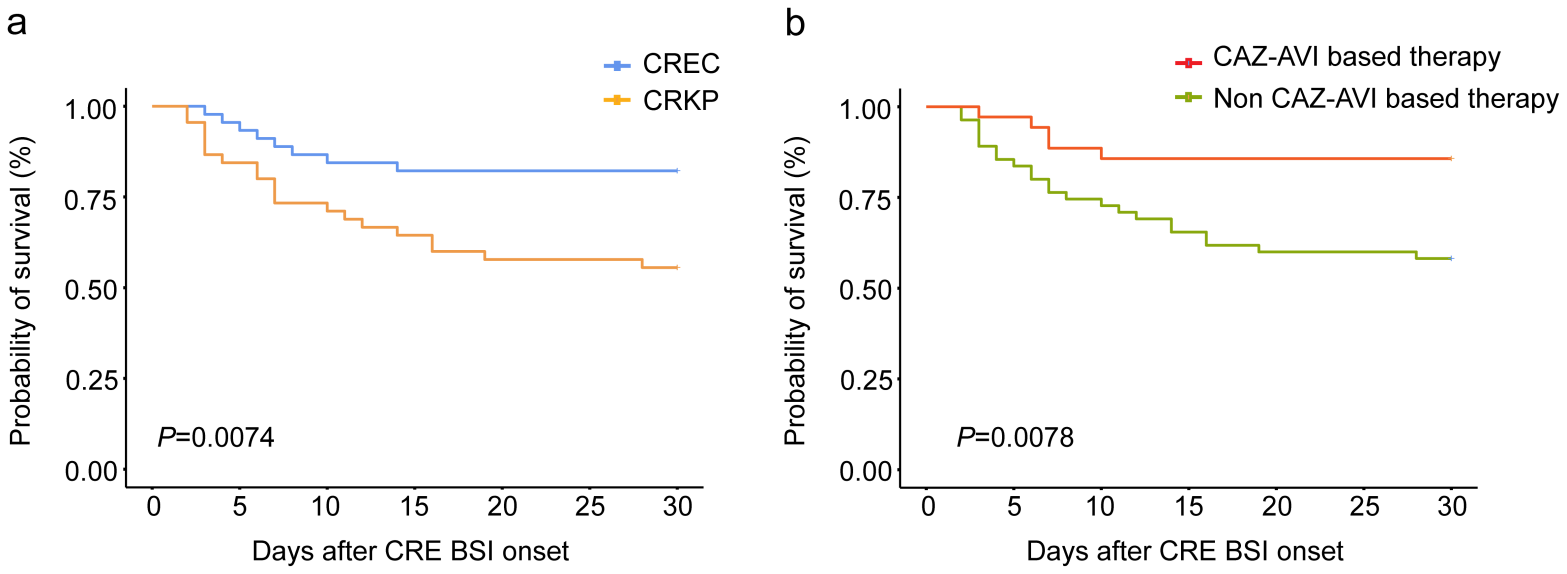
Kaplan-Meier curve of the 30-day survival probability of all patients with CRE BSI **(A)** patients with carbapenem-resistant Klebsiella pneumoniae (CRKP) or carbapenem-resistant Escherichia coli (CREC) bacteria. **(B)** patients with ceftazidime-avibactam (CAZ-AVI) based therapy or non-ceftazidime-avibactam-based (Non-CAZ-AVI) therapy.

Our comprehensive analysis sought to delineate the prognostic implications of various factors, including the specific strain of CRE and other clinical variables, on the mortality of hematological patients with CRE BSI. To neutralize the effects of host and ancillary confounding elements on patient outcomes, both univariate and multivariate analytical methods were employed over a 30-day observation period (**Table 3**). The initial univariate examination pinpointed several factors significantly correlated with patient prognosis. Notably, prior colonization by CRE within 90 days, BSI caused by CRKP, glucocorticoid use within 30 days prior to BSI onset, comorbidities unrelated to infection, bacteremia caused by other bacteria, multi-site infection preceding CRE BSI occurrence, treatment with CAZ-AVI-based therapy or combination therapy, and appropriate therapy were significantly associated with patient prognosis (p < 0.05). Subsequent multivariate analysis, adjusting for potential confounders, elucidated that BSI due to CRKP stood out as an independent risk factor adversely affecting survival (Odds Ratio [OR]: 4.805; 95% Confidence Interval [CI]: 1.2119–19.053; P = 0.029). Conversely, the administration of CAZ-AVI-based therapy was identified as an independent protective factor, significantly improving patient prognosis (OR: 0.128; 95% CI: 0.026–0.622; P = 0.013). Further stratification and survival analysis, utilizing the Kaplan-Meier method, compared the outcomes of patients with BSIs caused by CRKP against those with CREC infections, as well as the efficacy of CAZ-AVI-based therapy relative to treatments excluding CAZ-AVI. These analyses underscored a significantly reduced survival rate in patients with CRKP BSI compared to their CREC counterparts (P = 0.0074), as depicted in **Figure 3A**. Moreover, a marked enhancement in survival outcomes was observed for patients receiving CAZ-AVI-based therapy in comparison to those treated with alternative regimens (P = 0.0078) (**Figure 3B**).

**Table 3:** Univariate and Multivariate Analysis of thirty-day mortality for carbapenem-resistant Klebsiella pneumoniae (CRKP) and carbapenem-resistant Escherichia coli (CREC) bacteria.

Covariate	Odds Ratio (95% CI)	P Value	Adjusted Odds Ratio (95% CI)	P Value
Age	1.01 (0.97-1.04)	0.728		
CCI score < 2	2.48 (0.65-9.38)	0.181		
Prior 90-day CRE colonization	0.29 (0.1-0.82)	0.02	0.251 (0.061-1.029)	0.059
CRKP	3.7 (1.41-9.7)	0.008	4.805 (1.212-19.053)	0.029
Carbapenemases production	0.74 (0.24-2.29)	0.603		
Meropenem MIC of isolate>8ug/ml	1.22 (0.39-3.83)	0.733		
Imipenem MIC of isolate>8ug/ml	1.28 (0.44-3.71)	0.655		
Prior 30-day cephalosporins using	1.1 (0.44-2.74)	0.843		
Hematopoietic Stem Cell Transplantation	0.57 (0.21-1.53)	0.263		
Chemotherapy	1.06 (0.25-4.44)	0.936		
Prior 30-day glucocorticoids using	0.35 (0.14-0.89)	0.027	0.645 (0.185-2.251)	0.494
Comorbidity besides infection	3.12 (1.17-8.31)	0.022	3.075 (0.702-13.463)	0.140
Bacteremia caused by other bacteria	5.4 (1.61-18.11)	0.006	3.538 (0.8-15.64)	0.100
Multi-site infection before CRE BSI	3.12 (1.17-8.31)	0.022	1.902 (0.458-7.893)	0.379
Fungal infection	1.61 (0.51-5.05)	0.418		
Perianal infection	1.08 (0.43-2.76)	0.865		
Ceftazidime/Avibactam based therapy	0.23 (0.08-0.69)	0.008	0.128 (0.026-0.622)	0.013
Tigecycline-based therapy	0.8 (0.33-1.97)	0.627		
Combined therapy	0.32 (0.12-0.85)	0.022	0.934 (0.217-4.029)	0.927
Appropriate therapy	0.15 (0.04-0.64)	0.011	0.635 (0.091-4.449)	0.649
Hospitalization time before BSI	1 (0.99-1.01)	0.843		

CI, Confidence Interval; CCI, Charlson Comorbidity Index.

## 4 Discussion

The epidemiological landscape of CRE infections in patients with hematological malignancies exhibits significant geographical variability. While CRKP is identified as the principal CRE pathogen affecting this patient population in Europe and the United States, the scenario in China is markedly different. Here, both CRKP and CREC constitute nearly equal proportions of CRE infections among individuals with hematological diseases (Pouch and Satlin, 2016; Zhang et al., 2019). This parity is mirrored in our findings, which not only highlight a persistent rise in the incidence of CREC BSI but also establish these two pathogens as leading contributors to CRE BSIs within our clinical environment. Despite the increasing recognition of CRE as a critical threat to patients with hematological disorders, the comparative research focusing on the distinctions between infections caused by CRKP and CREC strains remains notably limited. Our study are committed to providing a comprehensive analysis of the clinical features, treatment responses, and outcome disparities in hematologic patients afflicted with BSIs caused by these two distinct CRE strains.

Our research has unveiled a concerning trend: hematological patients afflicted with BSI caused by CRKP exhibit a notably higher mortality rate compared to those with BSI caused by CREC. This disparity persists despite comparable infection rates and similar treatment modalities and clinical profiles between the two patient groups, with the exception of a more frequent occurrence of multi-site infections among those infected with CRKP. Further in-depth multivariate analysis reveals that CRKP infections independently amplify the mortality risk for these patients. Hence, we postulate that this phenomenon may be ascribed to the heightened virulence of CRKP strains compared to CREC strains. The emergence of carbapenem-resistant hypervirulent Klebsiella pneumoniae (CR-hvKP) strains, particularly noted in mainland China (Shankar et al., 2016; Krapp et al., 2017; Feng et al., 2018; Huang et al., 2018; Zhang et al., 2020), supports this hypothesis. CR-hvKP strains are distinguished not only by their rapid transmission within healthcare settings but also by their robust resistance to carbapenems, culminating in grave, sometimes fatal, outbreaks (Zhang et al., 2015; Gu et al., 2018). In our investigation, the virulence of CRKP strains and its correlation with mortality were not assessed through established experimental methodologies, such as the Galleria mellonella larvae infection model. This gap highlights the urgent need for future research to closely monitor the virulence and molecular characteristics of CRKP in BSIs, aiming to identify more effective treatments.

CAZ-AVI is a new antibiotic approved by the US Food and Drug Administration (FDA) in 2015 for treating CRE infections. Avibactam is a non-β-lactam β-lactamase inhibitor that exhibits activity against Ambler class A and certain class D carbapenemases, but not against class B metallo-β-lactamases (MBLs) (van Duin and Bonomo, 2016). Its efficacy in enhancing survival rates, achieving higher clinical success, reducing mortality, and minimizing toxicity in CRE infections has been well documented (Shields et al., 2017; van Duin et al., 2018; Chen et al., 2021; Karaiskos et al., 2021; Tumbarello et al., 2021). A pivotal study involving 577 patients with CRKP pneumonia highlighted CAZ-AVI’s effectiveness, showing a significant reduction in 30-day mortality rates when used alone or with other antimicrobials, compared to early non-CAZ-AVI regimens (Tumbarello et al., 2021). Further, (Shields et al., 2017). underscored CAZ-AVI’s superiority in treating CRKP bacteremia. Consistent with previous studies, our analysis, involving 35 patients administered CAZ-AVI therapy, revealed a notably reduced mortality rate (11.4%) compared to non-CAZ-AVI treatments (38.9%, P = 0.008), establishing CAZ-AVI as an independent factor for improved outcomes. Given its clear survival advantage, CAZ-AVI emerges as the recommended primary treatment for CRE BSI.

Understanding the geographical and institutional variation in carbapenemase distribution is crucial for combating CRE infections effectively. In China, the carbapenemase genes blaKPC and blaNDM are the most commonly identified in CRE strains, with CRKP strains largely producing KPC (77%) and CREC strains mainly producing NDM (75%) (Han et al., 2020). KPC, a serine-carbapenemase, is capable of breaking down aztreonam (ATM) but is vulnerable to the novel inhibitor avibactam. In contrast, NDM, a metallo-carbapenemase, does not affect ATM and exhibits resistance to avibactam (Logan and Weinstein, 2017). This differential enzyme activity and inhibitor susceptibility suggest that the CAZ-AVI and ATM combination offers a strategic therapeutic approach against MBL producers. While ATM maintains effectiveness against MBLs, its use alone is limited by the frequent occurrence of other resistance mechanisms, such as ESBLs and OXA-48, in MBL-producing Enterobacteriaceae (Marshall et al., 2017). Recent studies advocate for the use of CAZ-AVI alongside ATM to combat MBL producers (Davido et al., 2017; Shaw et al., 2018; Falcone et al., 2021). A notable prospective study demonstrated that patients with bloodstream infections caused by MBL-producing Enterobacteriaceae who received CAZ-AVI and ATM combination therapy experienced significantly lower 30-day mortality rates compared to those treated with other antimicrobials (Falcone et al., 2021). In our research, all tested CREC strains produced NDM, and a considerable portion of CRKP isolates also harbored the NDM gene. This prevalence underlined the efficacy of CAZ-AVI and ATM combination therapy, where a significant majority of treated patients achieved clinical success, leading to an impressively low mortality rate. Thus, identifying specific carbapenemase genes is pivotal for guiding therapeutic decisions. For facilities lacking the capability to perform detailed enzyme assays, particularly for identifying NDM in CREC strains, the recommendation is to favor CAZ-AVI plus ATM combination therapy, leveraging its proven success in addressing these challenging infections.

While CAZ-AVI has proven to be a significant advancement in treating CRE BSI, the increasing threat of antibiotic resistance emphasizes the need for exploring our arsenal of treatment options in diverse patient populations. In the pre-CAZ-AVI era, tigecycline was commonly used by clinicians to treat CRE infections. However, the debate over its clinical efficacy has persisted. Ni et al (Ni et al., 2016)conducted a systematic review and meta-analysis, which revealed that there were no significant differences in terms of overall mortality rate, clinical effectiveness rate, or microbiological effectiveness rate between the tigecycline group and other antibiotics (excluding CAZ-AVI). Exploring enhanced treatment strategies, such as combination therapy or high-dose regimens involving tigecycline, could potentially enhance its efficacy beyond standard single-agent therapies. Our investigation into the microbiological profiles of CRE strains disclosed that both CREC and CRKP variants show susceptibility to tigecycline, with CREC strains notably more sensitive than their CRKP counterparts. This observation aligns with findings from a comprehensive study by Zhang et al., which spanned 14 provinces and 25 tertiary hospitals, revealing a higher sensitivity of Escherichia coli to tigecycline compared to Klebsiella pneumoniae (Zhang et al., 2018). Further scrutiny within our study highlighted that patients treated with a tigecycline-based regimen experienced varying mortality rates, with those infected by CRKP facing a higher mortality rate (42.9%) in contrast to the CREC-infected group (25%). Although the difference in mortality rates did not achieve statistical significance, likely due to the small number of cases, it suggests that tigecycline-based treatments could remain a considerable option to reduce the occurrence of CAZ-AVI resistance for managing CREC BSI. However, hematological patients with CRKP BSI may benefit from early CAZ-AVI based treatment. These results provide novel insights for empiric treatment strategies or antibiotic stewardship policies of BSIs caused by different CRE strains.

The current research is subject to a few notable limitations. Firstly, it did not investigate the virulence factors of each strain, a critical aspect that could shed light on the microbiological underpinnings behind the observed higher mortality rates in patients with CRKP BSI. Secondly, due to substantial heterogeneity in treatment protocols for patients with CRE, it was not feasible to make meaningful comparisons between different antibiotic combinations. Thirdly, being a single-center retrospective study, our research is inherently constrained by the limitations associated with retrospective analyses, such as potential biases and the inability to control for all confounding variables. Consequently, there is a pressing need for more comprehensive studies that span a broader range of hospital settings to validate our findings and expand our understanding of the best practices for managing CRE infections.

## Data Availability

The original contributions presented in the study are included in the article/**Supplementary Material**. Further inquiries can be directed to the corresponding author.
