# Supplementary material for: A comparative analysis of clinical outcomes in hematological patients afflicted with bacteremia attributable to carbapenem-resistant Klebsiella pneumoniae versus Escherichia coli
Source: Front Cell Infect Microbiol. 2025 Jun 17;15:1600746. doi: 10.3389/fcimb.2025.1600746 (PMC12209189; doi:10.3389/fcimb.2025.1600746)
Supplement: Supplementary file 1 [file DataSheet1.docx]

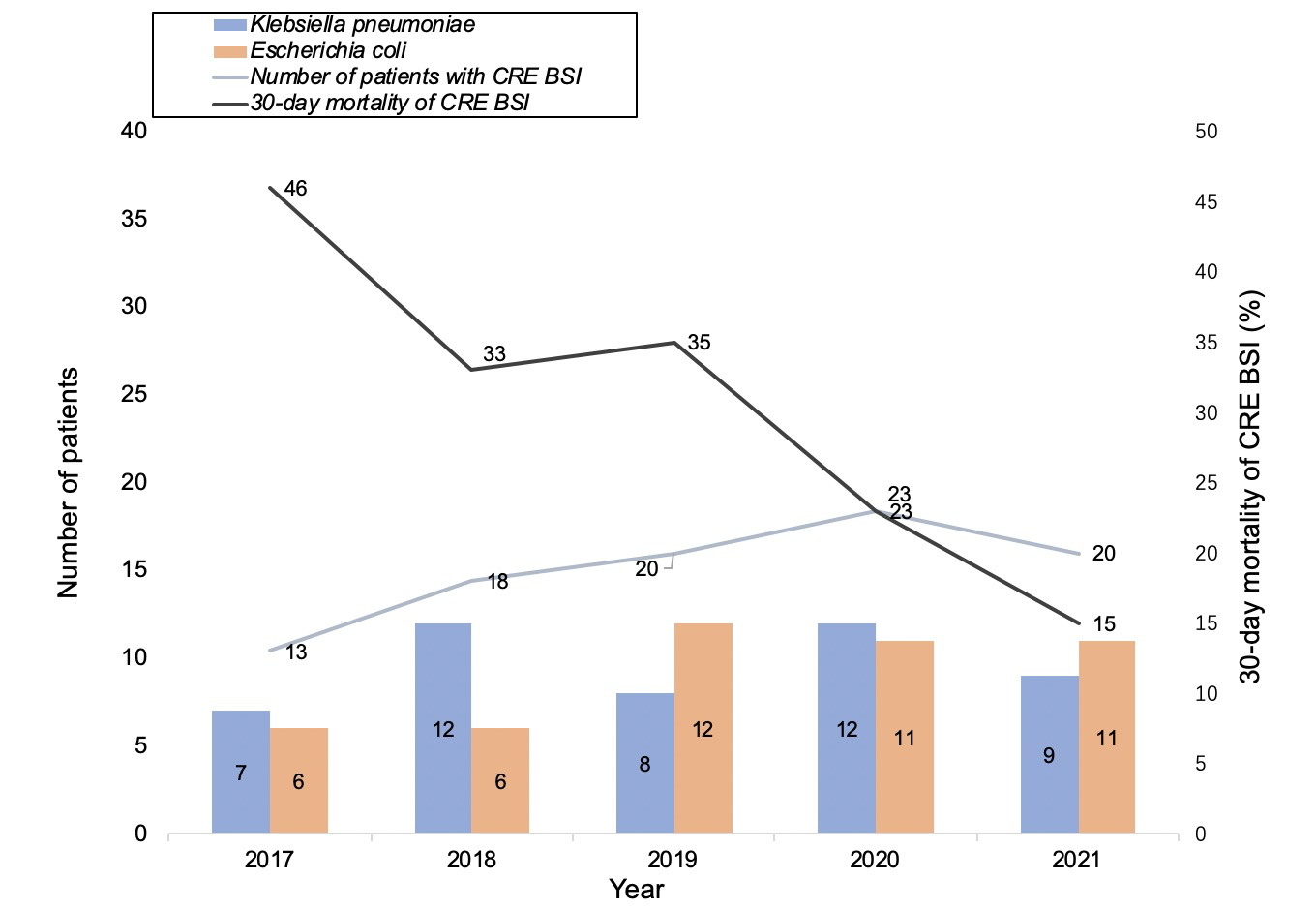


Figure S1 The annual number of patients and 30-day mortality of CRKP BSI and CREC BSI.

Abbreviations: CRE, Carbapenem-resistant Enterobacterale; BSI, bloodstream infection; CRKP, Carbapenem-Resistant *Klebsiella pneumoniae;* CREC, Carbapenem-Resistant *Escherichia coli.*


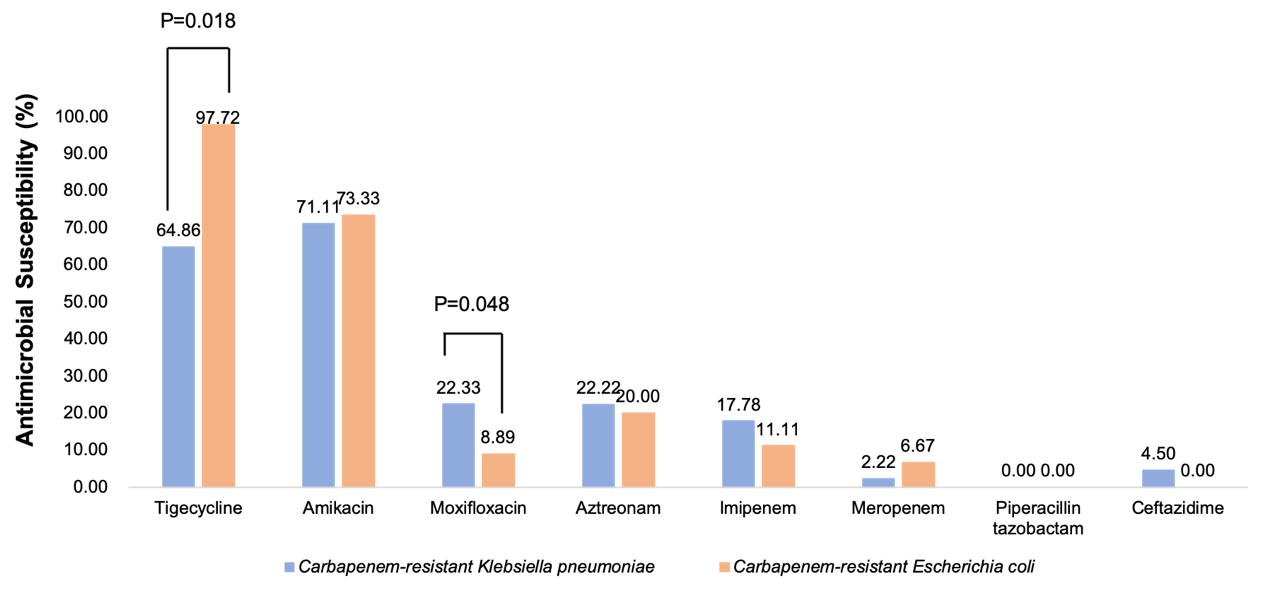


Figure S2 Comparison of susceptibility to prevalent antibiotics in patients with CRKP or CREC BSI.

Abbreviations: CRKP, Carbapenem-Resistant *Klebsiella pneumoniae;* CREC, Carbapenem-Resistant *Escherichia coli.*

Table S1 Identification of carbapenem resistance mechanisms in carbapenem-resistant blood isolates.

|  | Total （n=90） | CRKP(n=45) | CREC(n=45) |
| --- | --- | --- | --- |
| **Non-CP-CRE^a^** | 17 | 11 | 6 |
| **CP-CRE** | 66 | 29 | 37 |
| **Unknown^b^** | 7 | 5 | 2 |
| **Carbapenemase** |  |  |  |
| *Metallo-carbapenemase* | 36 | 11 | 25 |
| *Serine-carbapenemase* | 7 | 6 | 1 |
| **Carbapenemase genes** |  |  |  |
| NDM | 24 | 6 | 18 |
| KPC | 12 | 12 | 0 |
| Unkown | 9 | 3 | 6 |

^a^CRE does not produce carbapenemase.

^b^Due to limitations in 2017, it is unclear whether this CRE produces carbapenemase.

Table S2 Oligonucleotides used as primers for PCR in this study

| Target | Sequence (5’-3’) | Amplicon size (bp) | Reference |
| --- | --- | --- | --- |
| KPC | F-CGTCTAGTTCTGCTGTCTTG | 798 | [6] |
|  | R-CTTGTCATCCTTGTTAGGCG |  |  |
| NDM | F-GGTTTGGCGATCTGGTTTTC | 621 | [6] |
|  | R-CGGAATGGCTCATCACGATC |  |  |
